# Characterization of the Elastic, Piezoelectric, and Dielectric Properties of Lithium Niobate from 25 °C to 900 °C Using Electrochemical Impedance Spectroscopy Resonance Method

**DOI:** 10.3390/ma15134716

**Published:** 2022-07-05

**Authors:** Sevan Bouchy, Ricardo J. Zednik, Pierre Bélanger

**Affiliations:** 1 Piezoelectricity and Ultrasonics Technologies and Materials Laboratory at ÉTS (PULÉTS), Montréal, QC H3C1K3, Canada; sevan.bouchy.1@ens.etsmtl.ca (S.B.); pierre.belanger@etsmtl.ca (P.B.); 2 Department of Mechanical Engineering, École de Technologie Supérieure, Université du Québec, Montréal, QC H3C1K3, Canada

**Keywords:** lithium niobate, piezoelectricity, high temperature, impedance spectroscopy, resonance method

## Abstract

Lithium niobate (LiNbO${}_{3}$) is known for its high Curie temperature, making it an attractive candidate for high-temperature piezoelectric applications (>200 °C); however, the literature suffers from a paucity of reliable material properties data at high temperatures. This paper therefore provides a complete set of elastic and piezoelectric coefficients, as well as complex dielectric constants and the electrical conductivity, for congruent monocrystalline LiNbO${}_{3}$ from 25 °C to 900 °C at atmospheric pressure. An inverse approach using the electrochemical impedance spectroscopy (EIS) resonance method was used to determine the materials’ coefficients and constants. Single crystal Y-cut and Z-cut samples were used to estimate the twelve coefficients defining the electromechanical coupling of LiNbO${}_{3}$. We employed an analytical model inversion to calculate the coefficients based on a linear superposition of nine different bulk acoustic waves (three longitudinal waves and six shear waves), in addition to considering the thermal expansion of the crystal. The results are reported and compared with those of other studies for which the literature has available values. The dominant piezoelectric stress constant was found to be $e_{15}$, which remained virtually constant between 25 °C and 600 °C; thereafter, it decreased by approximately 10% between 600 °C and 900 °C. The elastic stiffness coefficients $c_{11}^{E}$, $c_{12}^{E}$, and $c_{33}^{E}$ all decreased as the temperature increased. The two dielectric constants $\epsilon_{11}^{S}$ and $\epsilon_{33}^{S}$ increased exponentially as a function of temperature.

## 1. Introduction

Many industries, including the aerospace and energy industries, employ critical infrastructure that operates at high temperatures, often reaching 600 °C or higher. Nondestructive testing (NDT) is required to ensure the integrity of these systems and thereby prevent the catastrophic failure of critical components that can lead to significant economic, environmental, and human losses. Thickness gauging using piezoelectric ultrasonic probes remains one of the most common measurements performed by NDT inspectors to evaluate corrosion and materials degradation. However, typical piezoelectric ceramics used in ultrasonic probes are limited to operations under 200 °C [1]. For example, petroleum refineries require periodic shutdowns (including lengthy cooling off to room temperature) for nondestructive testing (NDT); these shutdowns are costly endeavours, as they mean production interruptions and are followed by energy intensive restarting of industrial processes. A few solutions have been proposed in the literature to perform in situ thickness gauging at elevated temperatures, including employing waveguides that can be used to move the piezoelectric element away from heat sources [2], and air or water cooling systems [3]. Unfortunately, these solutions are bulky and difficult to install in small spaces or under insulation. There is therefore a need for alternative piezoelectric materials that can be used to manufacture small ultrasonic probes capable of withstanding high temperatures for extended periods of time.

Multiple potential piezoelectric candidates exist for high-temperature applications above 200 °C, including aluminum nitride (AlN), gallium orthophosphate (GaPO${}_{4}$), lanthanum titanate (LaTiO${}_{3}$), rare-earth oxyborates (ReCOB), and lithium niobate (LiNbO${}_{3}$) [4]. Of these candidates, lithium niobate is one of the most promising, because it combines a high Curie temperature of about 1210 °C with large piezoelectric coefficients [5,6,7] and is compatible with existing ultrasound technology [8,9]. Nonetheless, the experimental observation of piezoelectricity in LiNbO${}_{3}$ has been found to often degrade well below the Curie temperature [10,11,12,13]; this has recently been confirmed to be caused by internal shorting of the crystal from the apparition of electrical conductivity at elevated temperatures dominated by Li+ ion motion [14]. Although this phenomenon can be mitigated through the implementation of appropriate design strategies, practical application of LiNbO${}_{3}$ remains complicated by the poorly characterized material properties at elevated temperatures.

A large body of literature provides the elastic, piezoelectric, and dielectric coefficients of LiNbO${}_{3}$ at room temperature [15,16,17,18,19]. However, only a single study reports comprehensive material properties up to 500 °C [20], with only a select few material parameters being reported for higher temperatures [7,21,22,23]. The limited availability of high-temperature data is a consequence of experimental difficulties: high-temperature characterization requires placing a sample into a furnace for heating in a controlled atmosphere. Nassau et al. [7] used plunger rods to make contact with the sample inside the furnace; Tomeno et al. [23] bonded the sample to a rod to establish contact inside the furnace. Acoustic methods are traditionally used to extract various materials coefficients, however, as the crystal is not mechanically free, uncertainty as to the measured material parameters remains. This contrasts with electrochemical impedance spectroscopy (EIS) that only needs a mechanically unconstrained electrical contact. However, despite this advantage inherent in EIS, no study available in the literature provides a comprehensive set of material parameters up to 900 °C for LiNbO${}_{3}$. We therefore report the constant electric field elastic stiffness $c_{ij}^{E}$, the piezoelectric stress constant $e_{ij}$, the dielectric stress constant $\epsilon_{ij}^{S}$ (real and imaginary parts), and the electrical conductivity σ for LiNbO${}_{3}$ for 25 °C to 900 °C. This complete understanding of the material properties is indispensable for any high-temperature piezoelectric application of this promising material.

In this paper, the complete set of elastic and piezoelectric coefficients, as well as the complex dielectric constants and the electrical conductivity of LiNbO${}_{3}$ are provided from 25 °C to 900 °C and at atmospheric pressure. An inverse EIS approach using a 20 Hz to 20 MHz 500 mV excitation and an analytical model are used to determine the coefficients and constants of LiNbO${}_{3}$. The properties of LiNbO${}_{3}$ provided herein were obtained from Y-cut and Z-cut single crystal samples and compared with the literature, where available. An analytical model [22,24] was used to simulate the impedance spectrum through a linear superposition of nine possible bulk waves (three longitudinal waves and six shear waves) on an orthogonal basis. The complete set of material properties were then simultaneously extracted using an iterative best-fit method between the analytically modelled and experimentally observed impedance spectra.

## 2. Materials and Methods

### 2.1. Equations of Piezoelectricity and Electrochemical Impedance Spectroscopy Method

The electromechanical coupling of the piezoelectric effect is given in stress-charge form using the Einstein summation notation:

(1) $$\begin{array}{ccc} T_{ij} & = & c_{ijkl}^{E}S_{kl}-e_{kij}E_{k} \end{array}$$

(2) $$\begin{array}{ccc} D_{i} & = & e_{ikl}S_{kl}+\epsilon_{ij}^{S}E_{j}, \end{array}$$

where $T_{ij}$ (N/m${}^{2}$) is the stress tensor, $c_{ijkl}^{E}$ (C/m${}^{2}$) is the elastic stiffness tensor at constant electric field, $S_{kl}$ (unitless) is the strain tensor, $e_{kij}$ (C/N) is the piezoelectric tensor, $E_{k}$ (V/m) is the electric field, $D_{i}$ (C/m${}^{2}$) is the displacement field, and $\epsilon_{ij}^{S}$ (F/m) is the dielectric permittivity under constant strain [25].

Thermodynamics and crystal symmetry (3m point group) arguments impose that these various tensors exhibit a reduced number of independent coefficients. This means that the coefficients of the elastic (6 independent variables), piezoelectric (4 independent variables), and dielectric (2 independent variables) tensors referenced in Equations (1) and (2) can be summarized by the following matrix [26]:

$\begin{array}{ccc} & \;\;\;\;\;S\;\;\;\;\;\;\;\;\;\;\;\;\;\;\;\;\;\;\;\;\;\;\;\;\;\;\;\;\;\;\;\;\;\;\;\;\;\;E & \\ T\;\;\;\;\;\;D & \left(\begin{array}{ccccccccc} c_{11}^{E} & c_{12}^{E} & c_{13}^{E} & c_{14}^{E} & 0 & 0 & 0 & -e_{22} & e_{31}\\ c_{12}^{E} & c_{11}^{E} & c_{13}^{E} & -c_{14}^{E} & 0 & 0 & 0 & e_{22} & e_{31}\\ c_{13}^{E} & c_{13}^{E} & c_{33}^{E} & 0 & 0 & 0 & 0 & 0 & e_{33}\\ c_{14}^{E} & -c_{14}^{E} & 0 & c_{44}^{E} & 0 & 0 & 0 & e_{15} & 0\\ 0 & 0 & 0 & 0 & c_{44}^{E} & c_{14}^{E} & e_{15} & 0 & 0\\ 0 & 0 & 0 & 0 & c_{14}^{E} & c_{66}^{E} & -e_{22} & 0 & 0\\ 0 & 0 & 0 & 0 & e_{15} & -e_{22} & \epsilon_{11}^{S} & 0 & 0\\ -e_{22} & e_{22} & 0 & e_{15} & 0 & 0 & 0 & \epsilon_{11}^{S} & 0\\ e_{31} & e_{31} & e_{33} & 0 & 0 & 0 & 0 & 0 & \epsilon_{33}^{S} \end{array}\right) \end{array}$

                          Note: $c_{66}^{E}=\frac{1}{2}\left(c_{11}^{E}-c_{12}^{E}\right)$

The method used to extract the coefficients was the resonance method using electrochemical impedance spectroscopy (EIS) (also known as electrical impedance spectroscopy or electromechanical impedance spectroscopy) as a function of the intrinsic properties of the material, the geometry, and the density [25]. EIS is a well-established materials characterization technique [27] that can be used to extract multiple material parameter simultaneously.

In our experiment, we followed the method described in detail by De Castilla et al. [22,24]. For our samples, integrating the two constitutive piezoelectric Equations (1) and (2) over each sample face to satisfy the mechanical boundary conditions yielded a system of equations representing the displacement field with nine equations containing nine unknowns. The solution gave the expression of the unknowns as a function of voltage U(ω) (integration of the electric field), free electric charge *Q* (integration of the dielectric displacement over the sample volume), the angular frequency ω, and the material parameters ($c^{E},e,\epsilon^{S}$). For example, considering the electrical boundary conditions, we can integrate Equation (2) over the entire sample volume of size $2a_{1}\;\cdot \;2a_{2}\;\cdot \;2a_{3}$ [24]:

(3) $$\begin{array}{ccc} \int_{-a_{1}}^{a_{1}}\int_{-a_{2}}^{a_{2}}\int_{-a_{3}}^{a_{3}}\left(\begin{array}{c} D_{1}\\ D_{2}\\ D_{3} \end{array}\right)dx_{1}dx_{2}dx_{3}=e\int_{-a_{1}}^{a_{1}}\int_{-a_{2}}^{a_{2}}\int_{-a_{3}}^{a_{3}} & S & dx_{1}dx_{2}ydx_{3}+\epsilon^{S}.\int_{-a_{1}}^{a_{1}}\int_{-a_{2}}^{a_{2}}\int_{-a_{3}}^{a_{3}}\left(\begin{array}{c} E_{1}\\ E_{2}\\ E_{3} \end{array}\right)dx_{1}dx_{2}dx_{3} \end{array}$$

The electrochemical impedance Z(ω) of the piezoelectric material was obtained by taking the voltage ratio over the current I for a sinusoidal excitation with the frequency f=ω/2π [24]:

(4) $$\begin{array}{ccc} Z(\omega ) & = & \frac{U(\omega )}{-j.\omega .Q} \end{array}$$

At high temperature or high frequency, dielectric losses could also affect the impedance spectrum. Sometimes, an electrical conductivity may appear inside the sample due to ionic diffusion or other processes, especially at high temperature and low frequency. Then, the permittivity matrix has to be substituted with:

(5) ϵS=ϵS′−j.ϵS′′+σj.ω;−and−tanδS=ϵS′′ϵS′

where tanδ is the dielectric loss. The impedance is analytically described based on the elastic, piezoelectric, and dielectric properties, the geometry, and the density of LiNbO${}_{3}$. Therefore, the final step was to measure the experimental impedance spectrum and then solve the inverse problem by fitting this analytical solution to the experimental data over the temperature range [24]. Performing a best-fit regression of the analytical model to the entire measured electrochemical impedance spectrum by minimizing the cost function Equation (6) enabled the simultaneous extraction of the complete set of material parameters using all predicted resonant frequencies at once. As all data points of the spectrum (in our case, over 20,800 points for a single measured spectrum containing multiple resonances from 20 Hz to 20 MHz) were simultaneously taken into account, the material parameters were determined with unrivalled precision.

(6) $$Cost=\Sigma ((log(|Z_{model}\left|\right)-log(|Z_{meas}{\left|)\right)}^{2}+{\left(\phi_{model}-\phi_{meas}\right)}^{2})$$

### 2.2. Assumptions and Considerations

The following assumptions were made: the mass of the crystal is constant and the thermal expansion is linear with temperature. The dimensions and the density with thermal expansion can be expressed as:

(7) $$\begin{array}{ccc} t(T) & = & t_{0}.\left(1+\alpha_{ii}\left(T\right).\left(T-T_{0}\right)\right) \end{array}$$

(8) $$\begin{array}{ccc} A(T) & = & A_{0}.\left(1+\alpha_{11}\left(T\right).\left(T-T_{0}\right)\right).\left(1+\alpha_{jj}\left(T\right).\left(T-T_{0}\right)\right) \end{array}$$

(9) $$\begin{array}{ccc} \rho (T) & = & \frac{\rho_{0}}{t(T).A(T)} \end{array}$$

where the i=1 with j=3 represents a Y-cut crystal, and i=3 with j=1 represents a Z-cut crystal, $t_{0}$ is the thickness at 25 °C, t(T) is the thickness at temperature T, $\alpha_{ii}$ and $\alpha_{jj}$ are the thermal expansion coefficients, $T_{0}$ is the temperature at 25 °C, $A_{0}$ is the surface area of the samples at ambient temperature, A(T) is the surface area at temperature T, $\rho_{0}$ is density at ambient temperature, and ρ(T) is the density at temperature T. Sugii et al. [28] and Wong [29] measured the lattice parameters by X-ray diffraction (XRD). Congruent lithium niobate has a Li:Nb ratio of approximately 48.45:51.55. Note that a variation in the composition influences the physical properties of the material [30], as shown in Table 1.

For congruent LiNbO${}_{3}$, Abrahams et al. [6], Boysen et al. [31], and Wong et al. [29] agree on the lattice parameters to within ±0.01 Å from room temperature to 900 °C. Consistent with these three studies, our model used a lattice parameter that changed with temperature, as shown in Table 2.

### 2.3. Sample Preparation and Experimental Setup

The samples were congruent single-crystal LiNbO${}_{3}$ with the following dimensions: 0.500 × 10.0 × 10.0 mm${}^{3}$, and a density of 4640 kg/m${}^{3}$. The values extracted are very sensitive to the mass and geometry, so care was taken in determining those values [19]. As single-crystal lithium niobate is anisotropic, different orientations were required to extract the complete set of coefficients (elastic, piezoelectric, and dielectric). Nevertheless, due to the crystal symmetry with point group 3m, the combination of Y-cut and Z-cut orientations sufficed to extract all parameters of interest. The samples were coated using physical vapour deposition with a 20 nm titanium dioxide adhesion layer and then a 100 nm platinum electrode.

**Figure 1:**
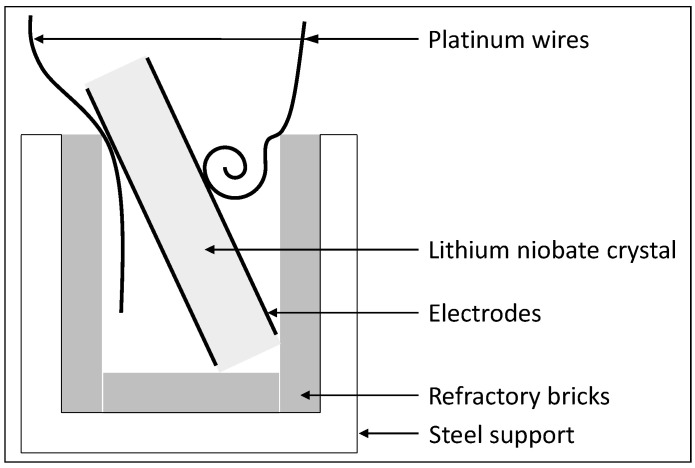
Schematic diagram of apparatus for performing impedance analysis at high temperature.

The sample was electrically connected with two platinum wires. Most acoustic-spectroscopy measurements sandwich the specimen between two transducers or bond the wires with paste, which constrains the specimen’s displacements, shifts the resonance frequencies from those at free vibrations, and also dampens the amplitude. Figure 1 shows that the specimen was therefore placed in a custom sample holder consisting of a U-shaped support brace, without external clamping forces, except for the specimen weight. This ensured a high reproducibility of the resonance-frequency measurements. A Keysight E4990A impedance analyzer was used to extract the EIS spectrum over a frequency sweep ranging from 20 Hz to 20 MHz, with a 500 mV excitation signal. For high-temperature characterization, the U-shaped support brace and the sample were placed inside a furnace, which heated the sample at 5 °C/min. Measurements were performed from 25 °C up to 900 °C in 50 °C increments, after which they were taken again during cooling from 900 °C down to 25 °C by 50 °C increments to see if the heating had changed the EIS spectrum of the sample. For each temperature step, a dwell time of 1 hour preceded all measurements to prevent temperature gradients in the sample and to avoid the potential appearance of any pyroelectric or thermoelectric effect. Our measurements were limited to temperatures of 900 °C or less due to experimental limitation of our apparatus.

## 3. Results and Discussion

### 3.1. Experimental Results

**Figure 2:**
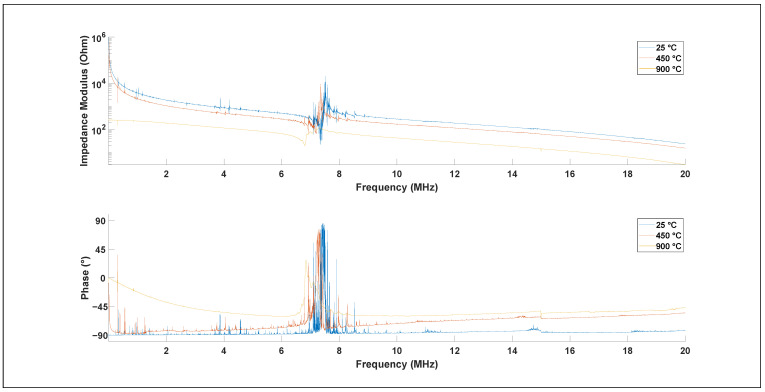
Electrochemical impedance modulus and phase spectrum of a Z-cut lithium niobate measured at 25 °C, 450 °C, and 900 °C.

Figure 2 shows the electrochemical impedance modulus and phase spectrum of Z-cut lithium niobate at representative temperatures of 25 °C, 450 °C, and 900 °C. The trend was consistent over the whole temperature range between 25 °C and 900 °C. A gradual shift of the longitudinal resonance frequency from 7.51 MHz to 7.30 MHz and a drop in amplitude from 20.0 kΩ to 117 Ω was observed for the longitudinal resonance between 25 °C and 900 °C, respectively. Furthermore, no differences were observed between measurements during the heating and the cooling of the sample, confirming the absence of damage. In addition, the temperature was held constant for one hour prior to each measurement to avoid any influence of a possible pyroelectric or thermoelectric effect, which can occur when there is a change of temperature or a temperature gradient in a sample.

**Figure 3:**
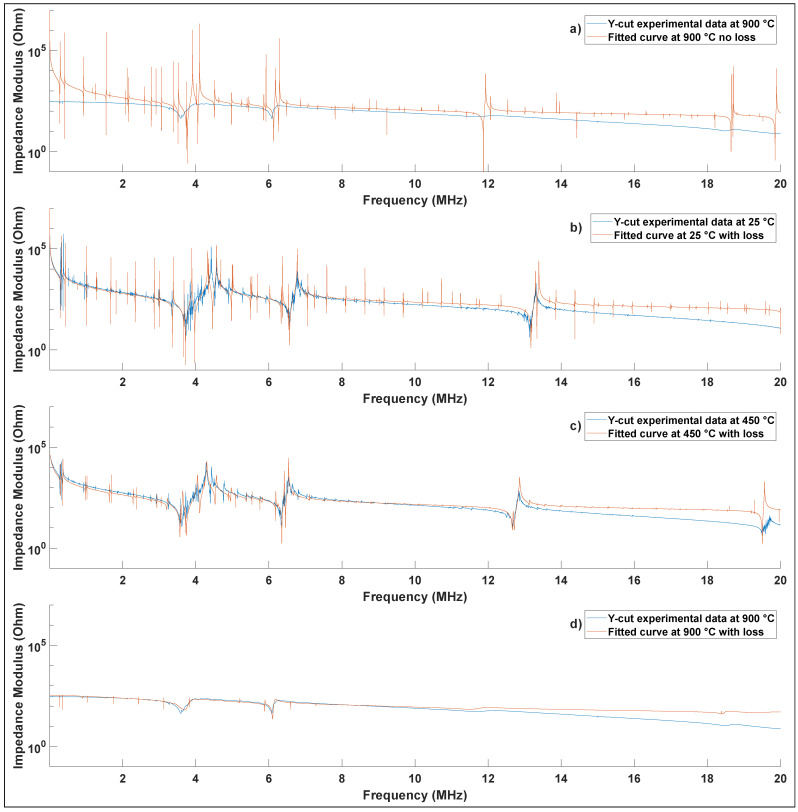
Electrochemical impedance modulus spectrum of a Y-cut lithium niobate with experimental data and fitted curve measured at: (**a**) 900 °C with no loss in the inverse model, (**b**) 25 °C with loss in the inverse model, (**c**) 450 °C with loss in the inverse model, and (**d**) 900 °C with loss in the inverse model.

Figure 3 shows the analytical model fitted to a representative experimental measurement of a Y-cut sample performed at 900 °C without taking into account loss, as well as the better fit accounting for loss at room temperature, 450 °C, and 900 °C. At room temperature for this Y-cut sample, the value of the cost function (Equation 6) is between 10,000 and 11,000 calculated over 20,800 points, regardless of whether losses are considered or not. However, at 900 °C, if we do not consider losses the value of the cost function soars to above 70,000. Alternatively, if we consider losses the value of the cost function is limited to 8900. Figure 3 therefore highlights the importance of taking into account the losses in the analytical model to extract the material parameters, particularly at higher temperatures.

A shift of the shear resonant frequency from 4.41 MHz to 3.99 MHz and a drop in amplitude from 130 kΩ to 230 Ω is observed for the shear resonance between 25 °C and 900 °C. Despite numerous harmonics and an extensive range, the analytical model accurately predicts the experimental observations over the entire frequency range. The two resonances at 282 kHz and 360 kHz correspond to radial mechanical modes: the first mode has two radial waves out of phase, whereas the second mode has radial waves in phase. The high frequency resonances at 4.4 MHz and 6.8 MHz are the shear and thickness modes, respectively. The analytical model correctly describes all these primary resonances, which enables an accurate extraction of materials parameters. In addition, other resonance peaks corresponding to higher-order harmonics of primary resonances are also well described.

Indeed, there are more higher-order resonances predicted by the analytical model than can be observed in the experimental data because the real sample is imperfect. When defects are present inside the material or the geometry is imperfect, some experimental resonances may exist, but they are too low in amplitude to be meaningfully detectable. However, one of the benefits of the EIS method is that the entire impedance spectrum is simultaneously used to extract all materials parameters, thus relying on many thousands of data points for each material parameter. Using the data from the Y-cut and Z-cut samples with the analytical model gives the complete set of elastic, piezoelectric, and dielectric coefficients in Table 3. The gradual increase of electrical conductivity at higher temperatures, particularly above about 500 °C, is consistent with the previously reported observations confirming the gradual appearance of ionic conduction caused by Li+ ion motion [14].

As is expected for any real material in the absence of a phase transformation or chemical reaction, the elastic stiffness coefficients are found to decrease with temperature. This is consistent with the crystal softening with increasing temperature, reducing the phonon velocity and the elastic stiffness. The dielectric permittivity also generally increases with temperature. Indeed, the thermal expansion of the crystalline structure allows for a more significant dielectric displacement, and thus a higher permittivity [32]. In addition, in a ferroelectric crystal such as lithium niobate, the dielectric dipoles become more mobile at higher temperatures and can better follow an external electric field, contributing to the dielectric permittivity. However, Das [33] highlights that a high coefficient of thermal expansion (CTE, >20 × 10${}^{-6}$/°C) or a negative CTE would impact and could be explained by phase changes or anisotropic strains. Although lithium niobate has a coefficient $\alpha_{33}$ that becomes negative at 700 °C, we do not see a phase change or anisotropic strains all the way up to 900 °C.

### 3.2. Literature Comparison

By combing the results obtained from Y-cut and Z-cut crystals and considering the dielectric loss, all electromechanical material properties can be readily extracted [26]. At high frequencies, minor higher-order harmonics appear and can be easily mistaken for noise. However, the analytical model predicts these higher-order harmonics, ensuring a stable fit to the experimental observations [22]. The coefficients measured in the present study are consistent with the values reported by Chen [20] and other authors in Table 4.

**Figure 4:**
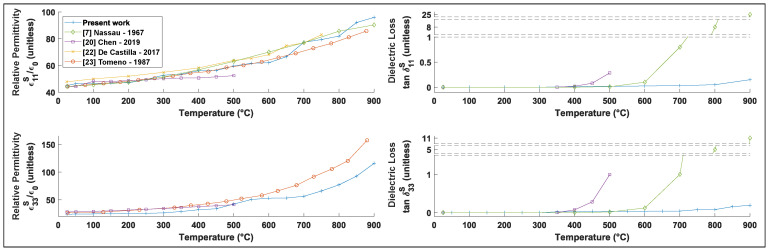
$\epsilon_{ii}$ dielectric constants at constant strain and loss $\tan\delta_{ii}$ in LiNbO${}_{3}$ as a function of temperature and comparison with the literature.

The relative permittivity follows an exponential law as a function of temperature [23]. Figure 4 presents a comparison with experimental data and shows that the extracted values are consistent with previous studies [7,20,22,23]. However, the model used in this article considers more parameters than previous models, in addition to the dielectric loss that was previously neglected. Thus, it is not surprising to find a slight discrepancy with the literature which used simpler models, as these additional parameters improve the reliability of the values extracted. Since the present model considers more coefficients and simultaneously fits the entire electrochemical impedance spectrum (as opposed to traditional models that only fit a single resonance at a time), the values deduced are more accurate and reproducible over the entire studied frequency range. Indeed, as our approach focuses on the shape of the curve as a whole, this facilitates high-temperature measurements, where the resonance peaks shift and broaden, making other methods less reliable.

**Figure 5:**
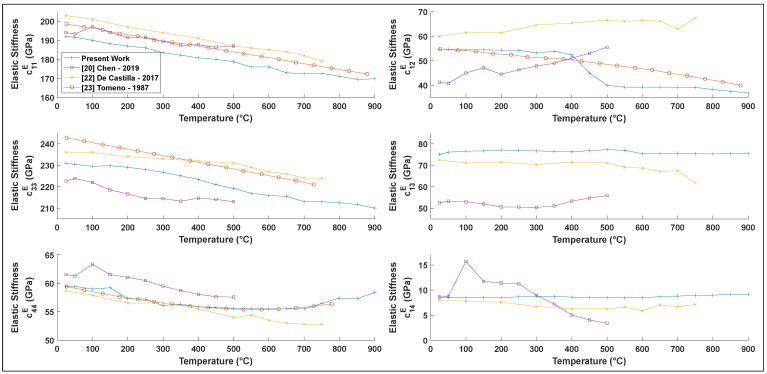
$c_{ij}^{E}$ constant electric field elastic stiffness in LiNbO${}_{3}$ as a function of temperature and comparison with the literature.

The temperature dependence of elastic compliance from room temperature to 900 °C was also determined (see Figure 5). The elastic stiffness coefficients $c_{11}^{E}$, $c_{33}^{E}$, and $c_{44}^{E}$ all generally follow the same trends as previously reported in the literature [20,22,23]. The slight differences could be explained by the method used, particularly in how the thermal expansion and loss were considered, as well as possible differences in sample composition. However, our results generally exhibit less scatter, which is expected given the large number of data points used to simultaneously extract each coefficient at each temperature.

**Figure 6:**
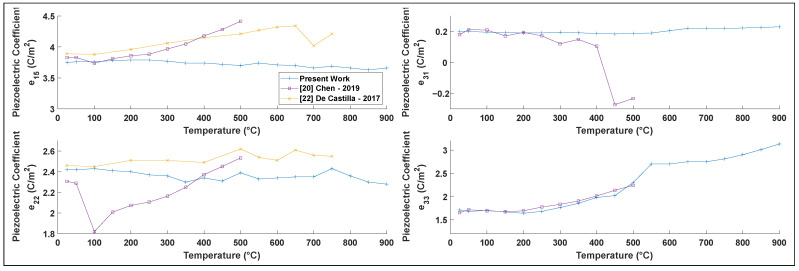
$e_{ij}$ measured piezoelectric stress constants as a function of temperature and comparison with the literature.

Only a few previous studies have determined the piezoelectric coefficients of lithium niobate at high temperatures [20,22] (see Figure 6). Our results are generally consistent with those previous studies, although our results exhibit noticeably less scatter. The coefficients $e_{15}$, $e_{22}$, and $e_{33}$ follow similar trends as those reported by other authors. However, we find that our value for $e_{31}$ disagrees with the one other reported literature value [20]; that study reported considerable scatter, including a change in sign of the parameter with no corresponding phase transition, suggesting a likely measurement or calculation error in that study.

## 4. Conclusions and Recommendations

We found that lithium niobate was a promising candidate for high-temperature piezoelectric applications up to at least 900 °C. Nonetheless, additional studies will be required to confirm the long-term stability of this material at high temperature to ensure no gradual degradation of the material properties; such long-term stability experiments would be the subject of an interesting follow-up study. However, we reported for the first time the complete set of twelve coefficients defining the electromechanical coupling of LiNbO${}_{3}$ from 25 °C to 900 °C at atmospheric pressure: elastic and piezoelectric coefficients, as well as complex dielectric constants, and the electrical conductivity. This was achieved by employing an analytical model inversion to calculate the coefficients based on a linear superposition of nine different bulk acoustic waves. This analytical model was used to simultaneously fit the entire electrochemical impedance spectrum between 20 Hz and 20 MHz obtained at each temperature. This method allowed for the consideration of dielectric loss and has the advantage of combining thousands of data points to calculate each material coefficient, thereby ensuring a high degree of precision and reproducibility.

## Figures and Tables

**Table 1:** Properties of lithium niobate according to Singh et al. [30].

Li:Nb	ρ	$\alpha_{11}$	$\alpha_{33}$
(unitless)	(g/cm${}^{3}$)	(10${}^{-6}$/ °C)	(10${}^{-6}$/ °C)
48.68:51.32	4.61	14.862	6.540
49.50:50.50	4.45	16.012	7.362

**Table 2:** Lattice parameter $\alpha_{ii}$ selected in this model depending on the temperature.

Temperature (°C)	25	47	67	87	107	200	300	400	500	600	700	800	900
$\alpha_{11}$ (10${}^{-6}$/ °C)	14.1	14.6	15.2	15.7	16.1	17.8	19	20.1	21	22	23	23.9	24.9
$\alpha_{33}$ (10${}^{-6}$/ °C)	4.1	4.2	4.3	4.3	4.3	4.0	3.5	2.7	1.6	0.4	−1.1	−2.8	−4.6

**Table 3:** Elastic, piezoelectric, dielectric constants, and electromechanical coupling factors for LiNbO${}_{3}$ from room temperature to 900 °C.

	25 °C	100 °C	200 °C	300 °C	400 °C	500 °C	600 °C	700 °C	800 °C	900 °C
Constant electric field										
Elastic stiffness (10${}^{10}$)	(N/m${}^{2}$)									
$c_{11}^{E}$	192	190	187	184	181	179	176	172	171	170
$c_{12}^{E}$	54.7	54.5	54.3	53.2	52.4	40.0	39.3	39.2	38.3	36.9
$c_{13}^{E}$	75.1	76.6	77.1	76.7	76.3	77.3	75.4	75.6	75.3	75.5
$c_{14}^{E}$	8.6	8.5	8.5	8.7	8.6	8.5	8.5	8.8	9.0	9.2
$c_{33}^{E}$	231	230	229	227	223	219	216	213	213	210
$c_{44}^{E}$	59.5	59.0	57.3	56.1	55.9	55.6	55.5	55.5	57.4	58.4
Piezoelectric stress										
constant	(C/m${}^{2}$)									
$e_{15}$	3.75	3.76	3.79	3.77	3.74	3.70	3.71	3.66	3.66	3.66
$e_{22}$	2.42	2.43	2.40	2.36	2.34	2.39	2.34	2.35	2.36	2.28
$e_{31}$	0.20	0.20	0.19	0.19	0.18	0.19	0.20	0.22	0.22	0.23
$e_{33}$	1.71	1.70	1.64	1.76	1.98	2.30	2.70	2.75	2.90	3.13
Constant strain										
Dielectric constant	(10${}^{-9}$ F/m)									
$\epsilon_{11}^{S^{\prime }}$	0.398	0.408	0.416	0.444	0.476	0.508	0.576	0.657	0.740	0.866
$\epsilon_{33}^{S^{\prime }}$	0.213	0.217	0.225	0.234	0.284	0.374	0.469	0.499	0.682	1.026
Dielectric loss	(unitless)									
$\tan\delta_{11}^{S}$	0.00002	0.00003	0.00007	0.0001	0.010	0.018	0.025	0.032	0.060	0.150
$\tan\delta_{33}^{S}$	0.00004	0.00005	0.0001	0.0001	0.017	0.034	0.036	0.041	0.079	0.190
Electrical conductivity	(10${}^{-3}$ S/m)									
σ	0.001	0.002	0.002	0.002	0.023	0.230	0.340	0.550	1.650	8.666

**Table 4:** Elastic, piezoelectric, and dielectric coefficients of LiNbO${}_{3}$ piezoelectric ceramic at room temperature with literature reference.

	Present Work	[5]	[15]	[16]	[17]	[18]	[19]	[22]	[20]
Constant electric field		Smith	Warner	Kovacs	Kushibiki	Ledbetter	Andrushchak	De Castilla	Chen
Elastic stiffness	(10${}^{9}$ N/m${}^{2}$)	(1971)	(1967)	(1990)	(1999)	(2004)	(2009)	(2017)	(2019)
$c_{11}^{E}$	192	203	203	198	199	200	199	203	194
$c_{12}^{E}$	54.7	57.3	53.0	54.7	54.7	55.3	54.7	59.9	42.8
$c_{13}^{E}$	75.1	75.2	75.0	65.1	68.0	67.7	70.0	72.5	52.2
$c_{14}^{E}$	8.6	8.5	9.0	7.9	7.8	8.7	7.9	8.0	8.4
$c_{33}^{E}$	231	242	245	228	234	235	240	236	223
$c_{44}^{E}$	59.5	59.5	60.0	60.0	59.9	59.5	59.9	58.7	61.5
Piezoelectric stress									
constant	(C/m${}^{2}$)								
$e_{15}$	3.75	3.76	3.7	3.69	3.66	3.65	3.67	3.89	3.85
$e_{22}$	2.42	2.44	2.5	2.42	2.41	2.39	2.38	2.46	2.30
$e_{31}$	0.20	0.23	0.2	0.30	0.33	0.31	0.34		0.18
$e_{33}$	1.71	1.33	1.3	1.77	1.89	1.72	1.60		1.66
Constant strain									
Dielectric constant	(10${}^{-9}$ F/m)								
$\epsilon_{11}^{S^{\prime }}$	0.398	0.392	0.390	0.404	0.398	0.399	0.389	0.423	0.389
$\epsilon_{33}^{S^{\prime }}$	0.213	0.247	0.257	0.233	0.236	0.232	0.247		0.248

## Data Availability

Not applicable.
